# Prebiotic Potential of Dietary Polyphenols in Colorectal Cancer Immunomodulation

**DOI:** 10.3390/foods14132392

**Published:** 2025-07-07

**Authors:** Bini Sreenesh, Elizabeth Varghese, Peter Kubatka, Samson Mathews Samuel, Dietrich Büsselberg

**Affiliations:** 1 Department of Physiology and Biophysics, Weill Cornell Medicine-Qatar, Education City, Qatar Foundation, Doha 24144, Qatar; bis4007@qatar-med.cornell.edu (B.S.); elv2007@qatar-med.cornell.edu (E.V.); sms2016@qatar-med.cornell.edu (S.M.S.); 2 Laboratory of Experimental and Clinical Regenerative Medicine, Small Animal Clinic, University of Veterinary Medicine and Pharmacy in Kosice, 04181 Kosice, Slovakia; peter.kubatka@uvlf.sk

**Keywords:** bacterial metabolites, colorectal cancer, gut microbiome, polyphenols, prebiotics

## Abstract

Molecular crosstalk between the gut microbiome and human diet represent a potential therapeutic avenue requiring further investigation as it can be applied to human health management and treatment. Colon cancer, the third leading cause of cancer mortality, is often linked to the gut microbiome. In vitro and in vivo studies and metagenomic research have revealed alterations in gut microbial flora among diseased individuals. The human diet is connected to these changes in microbial inhabitants related to the pathophysiology underlying colorectal cancer (CRC). Polyphenols are well-studied, naturally occurring plant secondary metabolites recognized for their anti-inflammatory and antioxidant properties. The anticancer activities of these compounds are increasingly reported, offering insights into the administration of these natural molecules for managing various types of cancer and developing novel medications from them. Recent investigations have highlighted the prebiotic-like effects of these compounds on gut microbial dysbiosis and their metabolism concerning colorectal cancer, influencing colon cancer by interfering with multiple signaling pathways. This review will focus on the existing literature regarding the prebiotic potential of dietary polyphenols, and further research in this area would be valuable, as the integration of artificial intelligence (AI) and machine learning (ML) can enable analysis of the connections between unique gut microbiome profiles and other dependent factors such as physiological and genetic variables, paving the way for personalized treatment strategies in gut microbiome-based health management and precision medicine.

## 1. Gut Health Duo: Pro- and Prebiotics

Probiotics and prebiotics are beneficial for host health management. Probiotics are living organisms administered to the host, while prebiotics are supplements that promote the selective growth or activity of beneficial microbial flora in the host. Probiotics have always been a component of health management due to their established positive effects in in vivo, in vitro, and clinical investigations, with their benefits attributed to their multi-dimensional mechanisms of action [1]. Evidence continues to emerge that probiotics and prebiotics influence the immune system and may enhance resistance to infections, particularly those of the gastrointestinal (GI) or respiratory tract, while also helping to mitigate allergies, especially in infants and young children. Evidence is gradually emerging for the potential use of probiotics and prebiotics to affect other gastrointestinal tract conditions, such as inflammatory bowel disease (IBD), irritable bowel syndrome (IBS), and colon cancer. Prebiotics are beneficial in enhancing calcium absorption, thereby promoting bone health [2]. Similarly, the role of prebiotics in modulating the brain-gut microbiome axis is also postulated, with an emphasis on a diet rich in polyphenols and supplementation [3]. Polyphenols, a diverse group of non-nutrient bioactive compounds with varying chemical structure, represent one of the largest families of phytochemicals found in plant-derived foods [4,5]. Polyphenols are gaining recognition as emerging prebiotics due to their potential to enhance gut health and alleviate intestinal dysbiosis [6]. Moreover, compared to traditional prebiotics that selectively enrich a particular target microbe, dietary polyphenols have been highlighted for their potential to interact with the gut microbiota, with their effects influenced by the food source, chemical structure, and individual variation in gut microbiota composition [7]. There is extensive research demonstrating the positive effects of probiotics and prebiotics on gastrointestinal tract infections, including IBD, IBS, and colon cancer. Probiotics and prebiotics confer health benefits via diverse mechanisms, notably by modulating the composition and activity of the gut microbiota, strengthening intestinal barrier integrity, and orchestrating host immune responses [8,9,10]. Many phytochemicals, particularly dietary polyphenols, have been shown to reduce CRC inflammation and impact the intestinal microbiome associated with CRC [11]. These compounds are subject to microbial biotransformation within the gut, resulting in the generation of bioactive metabolites that can modulate host physiological processes and immune function [12]. Previously, probiotics were thought to originate from food-derived microbes; however, recent advancements have shifted focus to microbiomes, with many newly identified gut microbial candidates proving to have probiotic potential. Furthermore, these microbial species can influence both gut microbial metabolism and the host’s metabolism, either directly or indirectly. Additionally, the gut microbial profile is unique and is sometimes transmitted from the maternal microbial reservoir [13]. Furthermore, the participants’ native microbial profiles significantly influence their response to probiotic or prebiotic interventions [14]. This is particularly relevant in CRC, where the effectiveness of microbiome-targeted strategies remains inconsistent, as often, CRC is associated with microbiome dysbiosis. The evidence supports the need for person-centered dietary supplements and tailored prebiotic-probiotic formulations to enhance beneficial bacterial species in prebiotic/probiotic-based disease management strategies. Such targeted gut microbial modulation through prebiotic intervention could be developed into a promising therapeutic approach for managing and preventing various gastrointestinal diseases, including CRC.

## 2. CRC, Microbiome, and Dysbiosis

**Figure 1:**
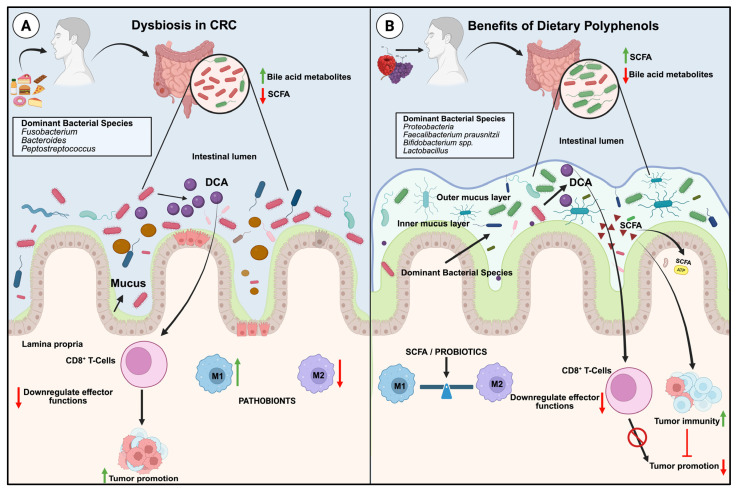
Interplay between gut microbiota, dietary intake and host epithelial immune integrity: (**A**) In CRC patients, gut barrier integrity is disrupted by pathobionts, microbiota derived deoxycholic acid (DCA), suppresses CD8+ T cell-mediated effector functions by impairing their cytotoxic activity and cytokine production, which contributes to reduced anti-tumor immunity and facilitate cancer progression. In early colorectal cancer, a high-fat diet, M1-driven colonic inflammation is enhanced due to butyrate depletion and pro-inflammatory bile acid accumulation. (**B**) Dietary supplementation with polyphenol-rich foods supports the enrichment of beneficial bacteria, which either reverse dysbiosis or promote the growth of beneficial gut microbial candidates. These Complex probiotics have been found to rebalance macrophage phenotypes; support improved mucosal barrier integrity and reduce colonic inflammation. Short-chain fatty acids (SCFAs) produced by the gut microbiome may improve tumor immunity, opposing the tumor-promoting effects caused by DCA-induced suppression of CD8+ T cell-mediated effector functions. Polyphenols and SCFA metabolites restore macrophage phenotypic balance. Created in BioRender. Sreenesh, B. and Samuel, S. M. (2025) https://BioRender.com/s0pw4gv (accessed on 3 July 2025).

These gut microbial interactions with cellular metabolism can be anti-cancerous or tumorigenic [15]. A balanced microbial composition with a healthy metabolism indicates good gut health, whereas a disruption of this balance signals the development of disease. This shift in microbial balance is known as dysbiosis [16]. Meta-genomic sequencing and other genomic techniques have indicated a shift in the gut microbiome profile in CRC patients [17]. Comparative 16S rRNA gene sequencing studies in a cohort of CRC patients with non-tumor tissues have indicated an evident decline in the genera *Ruminococcus* and an increase in the bacterial species *Fusobacterium* and *Bacteroides* [18]. A recent systematic review on microbiota dysbiosis and CRC revealed that the *Firmicutes* population contributed approximately 63.4% of the patient’s microbial load, whereas the value was 43.6% in healthy individuals. On the other hand, the predominant bacterial inhabitants in healthy individuals were proteobacteria, comprising 60.35% of the host microbiome, which decreased to 10.66% in the gut of CRC patients. Bacteroidetes were the second most abundant group, followed by Fusobacteria. Bacterial dysbiosis was evident at the species level, with reports of enrichment of *Peptostreptococcus* ten species in CRC patients and a reduction in numbers for *Flavobacterium* (*Flavobacteria*), *Pedobacter*, *Sphingobacterium (Sphingobacteria)*, *Caulobacter*, *Brevundimonas*, *Sphingomonas (Alphaproteobacteria)*, *Acidovorax*, *Janthinobacterium (Betaproteobacteria)*, *Buttiauxella*, *Rahnella*, *Acinetobacter*, *Psychrobacter*, *Pseudomonas*, *Stenotrophomonas (Gammaproteobacteria)*, and *Propionicbacterium (Actinobacteria)* [19]. Studies have confirmed the interplay between CRCs and gut microbiota dysbiosis (Figure 1), and the bacterial population can positively or negatively influence CRC development and progression. Certain bacterial species, notably *Fusobacterium nucleatum*, have been associated with colorectal carcinogenesis through multiple mechanisms, including alterations in host immune responses, activation of pro-inflammatory signaling pathways, and stimulation of tumor cell proliferation [20]. *Alloprevotella* is a cancer biomarker often reported to be inversely associated with cytokines such as TNF-α, IFN-γ, and CXCR4. *Alloprevotella*, a bacterium enriched in CDR tissues compared to normal intestinal mucosa, has been reported as a producer of short-chain fatty acids (SCFAs) that may modulate immune cell function and reduce the risk of CRC. On the other hand, the enrichment of *Megamonas* was evident in CRC patients harboring KRAS mutations [21].

Immune cells play a crucial role in the development and progression of CRC and are closely linked to the gut microbiome. A substantial bacterial infiltration was evident in colonic tissue compared to healthy tissue. The crosstalk between these bacterial components and mucosal immunity led to the recruitment of antibacterial neutrophils, which account for the presence of neutrophils in healthy peritoneal tissue. In contrast, patients with peritoneal metastasis were missing these recruited neutrophils [10]. This, in turn, highlights the importance of mucosal integrity and the remodeling of the tumor microenvironment (TME) in the management and treatment of CRC. M1 and M2 macrophages act as key modulators of the host immune system against tumors in a location-dependent, mutually interconnected manner [11,18,19]. In acute inflammation, monocyte infiltration modifies the tight junctions, followed by the M1 phenotype, which contributes to epithelial barrier disruption [22]. The probiotics were found to downregulate M1 polarization further, thereby enhancing M2 polarization factors and maintaining a balance in macrophage phenotype (Figure 1). This is beneficial for improving mucosal barrier function and reducing gut microbial inflammation [23]. In this context, probiotics may help influence the TME by promoting beneficial species and decreasing the reported CRC marker candidates [24]. Short-chain fatty acids (SCFAs), particularly butyrate, are key microbial metabolites produced through the fermentation of dietary fiber by the gut microbiota and are essential for maintaining colonic homeostasis. Butyrate functions as the principal energy substrate for colonocytes and exerts anti-inflammatory effects by inhibiting histone deacetylases (HDACs), thereby regulating gene expression and facilitating the differentiation of regulatory T cells [25]. Evidence suggests that SCFA butyrate could upregulate the immune functions of CD8+ T cells in contrast to the suppressive effects of bacterial bile acid metabolites (Deoxycholic acid) [26] (Figure 1). Human gut microbiota and its metabolites like SCFAs, Vitamins, amino acids and lipids are known to play an essential role in maintaining good gut health. It has also been reported that microbial dysbiosis is frequently associated with CRC. Dysbiosis is characterized by an abundance of cancer-promoting bacteria in comparison to protective bacteria. As a result of the interactions between these cancer-promoting gut microbes and their metabolites, further increases in mucosal membrane permeability occur, which in turn promote the exposure of host cells to more carcinogens, thereby worsening the inflammation [22]. A promising therapeutic strategy to mitigate this pathological condition involves restoring microbial homeostasis by enhancing the abundance of beneficial probiotic species while concurrently reducing pathogenic bacterial populations. This microbiota-targeted approach has demonstrated potential in the treatment and management of both CRC and IBD [27]. Dietary polyphenols have shown substantial potential to modulate the gut microbiota by promoting the proliferation of beneficial genera, including *Bifidobacterium* and *Lactobacillus*, while simultaneously suppressing pathogenic taxa such as *Clostridium* and *Escherichia coli*. This selective modulation of microbial communities supports the re-establishment of intestinal homeostasis and is critically implicated in lowering the risk of CRC [28]. The association between colorectal tumorigenesis and microbial dysbiosis has long been a topic of controversy. The evidence indicates that gut microbiota may contribute to tumorigenesis, CRC modulation, and therapy [18]. In vivo studies have demonstrated that the administration of polyphenols restores the microbial profiles of diseased animals to those of healthy controls, indicating the potential of polyphenols to combat colitis and dysbiosis [27]. If this is the case, these polyphenol supplements can act as a prebiotic component to the beneficial gut microbiota through diet, thereby revealing their potential as a potent immunomodulator in CRC. These bioactive compounds may play a protective role by regulating the tumorigenic bacterial population and selectively enriching the beneficial anti-cancerous microbiota. Despite these promising findings, the precise mechanisms underlying the selective modulation of microbial populations remain poorly understood.

## 3. Dietary Polyphenols and Their Gut Microbial Metabolites in CRC

Significant research studies have demonstrated that the gut microbiome plays a crucial role in polyphenol metabolism, and these metabolites offer numerous health benefits to humans [27]. Owing to their complex chemical structures and high molecular weights, polyphenols are poorly absorbed in the small intestine and primarily transit to the colon, where they undergo extensive microbial metabolism. In this compartment, the gut microbiota converts them into low-molecular-weight phenolic metabolites with enhanced bioavailability, which can be readily absorbed and exert beneficial effects on host health [29]. A diet rich in prebiotics and reduced antibiotic use may support gut microbial metabolism and overall gut health [30]. Furthermore, it is proposed that gut microbes may metabolize these bioactive compounds to produce novel metabolites, which can also benefit the host. In an earlier study using gnotobiotic mice, it was proven that gut microbiota aids the bioconversion and bioavailability of phenolic metabolites from plant-derived polyphenols [31]. The biotransformation of polyphenols by the gut microbiota not only improves their bioavailability but also critically contributes to shaping the composition and functional activity of the gut microbial community, thereby exerting downstream effects on host physiological processes and immune regulation [32,33]. The emerging evidence warrants including metabolomics analysis alongside gut microbial analysis to enhance the efficacy of cancer treatment regimens, with a focus on microbiota-based therapies [34]. Evidence suggests that a diet rich in fruits, vegetables, and fiber, such as the Mediterranean diet (MD), is often inversely linked to CRC risk and mortality. In contrast, an increase in CRC risk is associated with Western dietary patterns [35]. Epidemiological evidence indicates that strong adherence to the MD is associated with a markedly reduced incidence of CRC, pointing to the protective effects conferred by this dietary pattern [36,37,38]. A study conducted by Semmelweis University demonstrated the protective role of the MD, especially in Hungary, where the CRC rate is alarmingly high [37]. The in vivo experiments in gnotobiotic mice have demonstrated the efficacy of polyphenols in alleviating colitis and inhibiting CRC. Additionally, it has been established that these dietary compounds can reverse disease-induced dysbiosis by enriching probiotics and reducing pathogenic and pro-inflammatory microbes [27]. Polyphenol-induced modulation of the gut microbiota supports the preservation of intestinal homeostasis and is associated with a reduced risk of CRC [39]. Numerous dietary polyphenols and their metabolites have undergone clinical studies. They are established as treatment options in specific cancer treatment regimens, either alone or in synergy with conventional anti-cancer drugs. Here, we highlight several compounds that have been extensively investigated, including clinical research that unveils their modes of action.

### 3.1. Short-Chain Fatty Acids (SCFAs)

**Figure 2:**
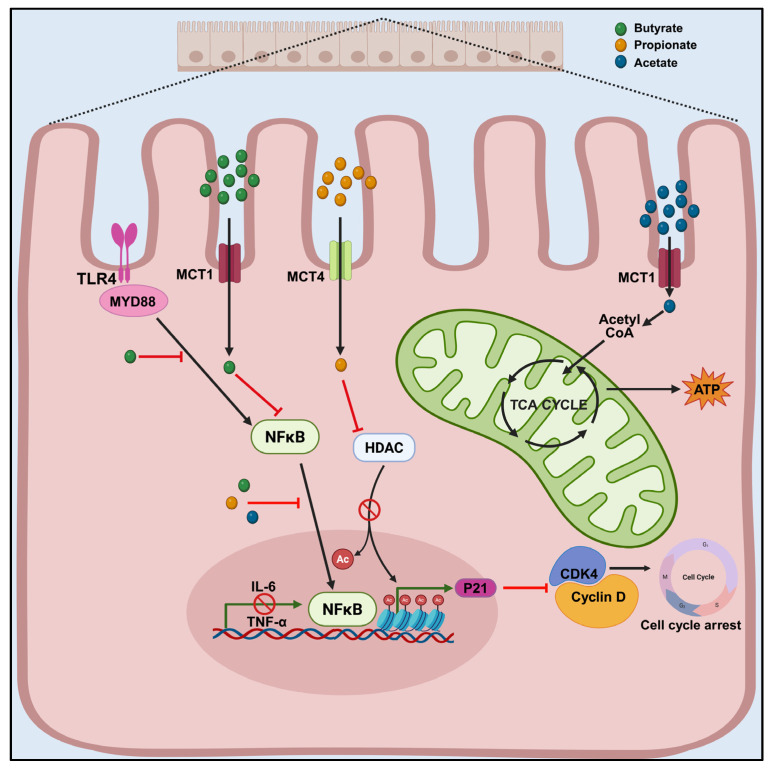
SCFA-mediated regulation of inflammation, gene expression, and metabolism in colorectal cancer: Bacterial metabolites Short-chain fatty acid (SCFA) butyrate can inhibit Toll-like receptor 4 (TLR4) expression in epithelial cells. TLR4 is a receptor involved in immune response to bacterial lipopolysaccharides (LPS), which in turn inhibits TLR-induced myeloid differentiation factor 88 (MyD88). By suppressing MyD88 recruitment and downstream signaling, the pathway effectively blocks the activation of the nuclear factor kappa B (NF-κB) signaling cascade. It reduces the transcription of pro-inflammatory genes (IL-6, TNF-α). Similarly, SCFAs (butyrate and propionate) enter cells via monocarboxylate transporters (MCTs) and inhibit histone deacetylase (HDAC), which in turn upregulate the expression of the tumor suppressor gene P21, leading to cell cycle arrest. Butyrate and acetate are converted to acetyl-CoA, while propionate is converted to succinyl-CoA, which enters the TCA cycle to produce ATP, promoting colonocyte health and energy balance—a critical factor in CRC prevention. Created in BioRender. Sreenesh, B. and Samuel, S. M. (2025) https://BioRender.com/9l3ls12 (accessed on 3 July 2025).

Short-chain fatty acids are the primary microbial metabolites produced by gut microbes during polyphenol metabolism. The depletion of these probiotic metabolites is often linked to carcinogenesis [40]. The relative abundance of different types of fatty acids depends on the gut’s microbial inhabitants, with acetate, propionate, and butyrate being the primary short-chain fatty acids (SCFAs) available. This is because the bacteria that produce acetate are more numerous than those that produce butyrate and other SCFAs. Major butyrate producers are anaerobes, so butyrate production is higher in the lumen [34]. Nearly 95% of these short-chain metabolites are absorbed by colonocytes and utilized for energy production, which is essential for maintaining a healthy epithelial lining and bolstering the immune system [41]. This supports normal cell energy production, keeps the gut barrier, and suppresses inflammation in healthy individuals. However, in the diseased state, butyrate production and concentration in the lumen are reduced due to microbial dysbiosis, leading to metabolic shifts, impaired barrier function, and inflammation [42]. Microbial-derived SCFAS are known to activate G protein-coupled receptors (GPRs) GPR 41/43 and can inhibit (Figure 2) TLR2, 4, 6, and 9 induced MyD88 (Myeloid differentiation factor 88) [23]. GPR43 is a G-protein-coupled receptor reported to be present in the colon and intestine. SCFAs can act as ligands for these receptors, inducing an antitumor effect by causing G0/G1 cell cycle arrest and apoptosis. Activation of GPR43 by SCFAs is crucial for the proper resolution of inflammation in diseased mouse models, and mutant mice lacking GPR43 receptors demonstrate impaired ability to resolve inflammation in experimental models [42] A high butyrate content is beneficial for human health and is believed to serve as a biomarker for prebiotic effects [7]. Additionally, a high butyrate content is associated with a reduced risk of intestinal tumor formation [43]. SCFAS creates an acidic environment within the lumen, inhibiting the growth of other pathogenic microbes. Furthermore, these SCFAs serve as nutrient supplements to other beneficial microbes [34]. SCFAs also regulate carcinogenesis by inhibiting HDACs, thereby preventing cell division [44,45]. Butyrate accumulates and acts as an inhibitor of HDACs, as they are generally not metabolized in cancer cells due to the Warburg effect [46].

The cyclin-dependent kinase inhibitor p21 waf1 regulates the cell cycle by interacting with G1 cyclin/CDK complexes [44]. The inhibition of HDAC activity by butyrate enables the recruitment of transcription factors Sp1 or Sp3, which leads to the enhanced expression of p21Waf1/Cip1, which in turn arrests the cell cycle (Figure 2) via a p53-independent pathway [45]. Downregulation of HDACs can lead to elevated cell apoptosis and cell cycle inhibition in colon cancer [47]. Sodium butyrate is reported to enhance apoptosis and limit the proliferation rate in Huh-7 hepatocarcinoma cells overexpressing intracellular microRNA miR-22, which results in increased reactive oxygen species (ROS) [48]. In this way, these molecules play a vital role in maintaining the TME, and their concentration is reported to be lower in patients with CRC. The microbial fermentation product propionate may induce an antitumor effect [49]. Butyrate suppresses inflammation by maintaining mucosal integrity in the colon via upregulation of regulatory T Cells [50]. SCFAs accumulated by microbial metabolism act as a precursor for energy generation in colonocytes via TCA cycle, which facilitates the maintenance of good intestinal health (Figure 2). Data mining has revealed the investigations regarding synergetic effects of butyrate in combination with existing chemotherapeutic agents in vitro and in vivo [34].

### 3.2. Resveratrol

**Figure 3:**
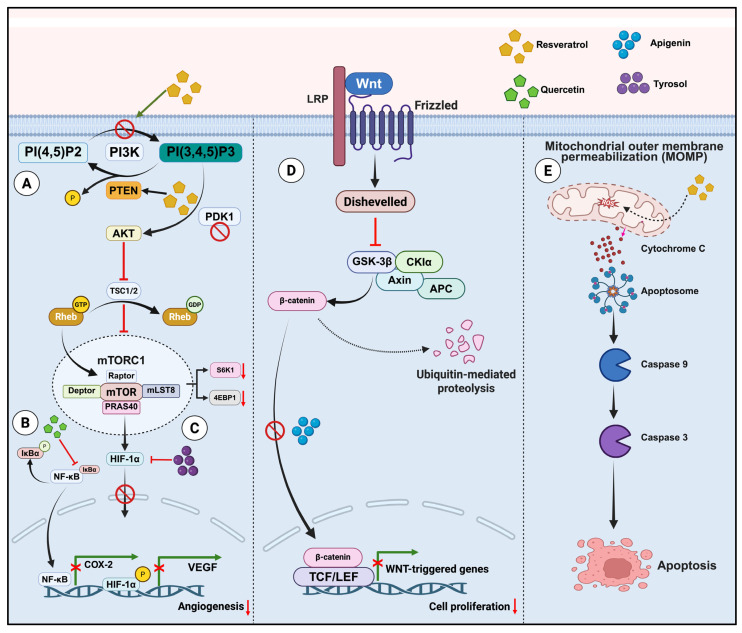
Molecular Mechanisms of Polyphenol-Mediated Regulation of Cell Proliferation, Inflammation, and Apoptosis in Cancer pathways: (**A**) Dephosphorylation of PTEN downregulates PIP3, which in turn downregulates the AKT pathway, reducing cell proliferation and promoting apoptosis. The downregulation of p-AKT upregulates TSC2, which deactivates Rheb, ultimately inhibiting mTORC1 and downregulating its downstream targets. This process inhibits protein synthesis and cell growth (**B**) Molecules that inhibit NF-κB prevent IκB-α phosphorylation and ubiquitin-mediated proteasomal lysis, allowing the binding of the cytoplasmic inhibitor IκB-α to NF-κB dimers. This action prevents NF-κB translocation into the nucleus, resulting in the transcriptional activation of inflammatory genes. (**C**) Tyrosol, a natural phenolic compound primarily found in olive oil, inhibits HIF-1α, which in turn down regulates Vascular endothelial growth factor (VEGF), leading to reduced angiogenesis and suppression of tumor progression (**D**) Apigenin suppresses the activation of β-catenin/TCF/LEF signaling by blocking the nuclear translocation of β-catenin. This inhibition leads to the downregulation of target genes, thereby inhibiting cell proliferation and survival. (**E**) Resveratrol-induced ROS generation results in mitochondrial membrane depolarization and the opening of mitochondrial permeability transition pores, which leads to the release of cytochrome C into the cytoplasm. This process activates caspases, promoting apoptosis. Created in BioRender. Sreenesh, B. and Samuel, S. M. (2025) https://BioRender.com/f368mxv (accessed on 3 July 2025).

Resveratrol is a naturally occurring polyphenol from the Stilbenes group known for its anticancer activity. Resveratrol exerts anticancer effects by inhibiting PI3K/AKT signaling pathway through the dephosphorylation of PTEN, which in turn induces apoptosis in colon cancer cells (Figure 3) [51]. Researchers have recognized its to chemo sensitize the TNF-β-induced survival of 5-FU-treated colorectal cancer cells [52]. Resveratrol treatment in HCT 116 and SW620 showed an increased intracellular ROS level, which may increase the permeability of the mitochondrial membrane and release of cytochrome C (Figure 3). Moreover, supporting these findings, resveratrol treatment has shown an elevated level of expression in Bax, cytochrome c, cleaved caspase-9, and cleaved caspase-3. The study postulates that the activation of the ROS-mediated mitochondrial pathway may account for the resveratrol-induced apoptosis in CRC cells [53]. Various studies have reported that resveratrol modulates cancer cell proliferation by downregulating CDK4/Cyclin D1, thereby facilitating the induction of P21 and P53 [54]. Gut microbiota is often associated with resveratrol metabolism in the gut [55]. This small-molecular-weight natural polyphenol undergoes biotransformation in the liver and intestine via reactions such as glucuronidation and sulfation [56]. These molecules are metabolized in the large intestine, which often increases their bioavailability and transforms them into more valuable metabolites [55]. Microbial biotransformation can convert the low-availability glucoside piceid into resveratrol [57]. Researchers have reported that the gut microbiome of Bacillus cereus is involved in this biotransformation [50]. The antioxidant activity of these microbial metabolites exceeds that of resveratrol, and their molecular targets are similar to those of resveratrol, including SIRT1 and NF-κB [55].

### 3.3. Ellagitannins

Ellagitannins are bioactive phenolic compounds found in berries, which are highly metabolized by gut microbiota [38]. Ellagitannins from black raspberry and their derivatives (ellagic acid, urolithin A (UA), and urolithin B) have demonstrated potential anticancer activity in HT-29 colon cancer cells by regulating the cell cycle (upregulation of p21) and apoptosis signaling pathways, as confirmed by the activation of caspase 3 and the cleavage of poly (ADP-ribose) polymerase (PARP) [58]. Gut microbial metabolism of dietary ellagitannins involves the cleavage of its lactone ring followed by its decarboxylation and dihydroxylation reaction, resulting in the formation of various microbial metabolites like pentahydroxy urolithin, tetrahydroxy- (urolithin D, urolithin E and urolithin M6), trihydroxy- (urolithin C and urolithin M7), dihydroxy- (urolithin A and iso urolithin A), and monohydroxy- (urolithin B), dibenzopyran-6-one metabolites [59]. UA has been well studied and documented as an emerging natural anticancer agent. An in vitro investigation of UA in HT29 has demonstrated elevated levels of p-ERK and p-AMPK, whereas in SW620 there was a significant reduction in the expression levels of phosphorylated AKT and mTOR. On the other hand, tumor suppressors p-c-RAF and p-PTEN were significantly upregulated, shedding light on the possible involvement of UA in the p-c-RAF/MEK/p-ERK signaling pathway. The study also highlights the potential involvement of these molecules in the PI3K/p-AKT/mTOR pathway and explains the underlying mechanism in metastasis [60].

### 3.4. Hydroxytyrosol and Tyrosol

Olive oil is the primary culinary fat in the MD, and the phytochemicals in olive oil are beneficial in managing CRC. The well-characterized polyphenols present in olive oil include tyrosol (Tyr), hydroxytyrosol (HT), oleuropein (Ole), and its aglycone [61,62]. The antitumor activity of tyrosol has been established through both in vitro and in vivo experiments, and its role in reducing reactive oxygen species and inflammatory cytokines in MC38 cells has also been demonstrated. Furthermore, western blot analysis has revealed its possible mode of action by inhibiting the activation of the HIF-1α/NF-κB signaling pathway (Figure 3) [63]. HT could downregulate EGFR (epidermal growth factor) expression in the HT29, CaCO_2_, and WiDR colorectal cell lines, and it is proposed that their mechanism of action involves lysosomal and proteasomal degradation. HT was able to induce phosphorylation of EGFR at PY1045 and also activate ubiquitin ligase Cbl [64]. Before considering these dietary compounds as cancer preventives or anticancer drugs, it is highly recommended to address compound-specific research on nutritionally relevant concentrations and the potential influence of these compounds on chemotherapy metabolism [65].

### 3.5. Apigenin

Apigenin is a well-studied, naturally occurring plant-derived flavone that has been extensively researched for its anti-tumorigenic activity among dietary components and is abundantly present in common fruits and vegetables [66,67]. Apigenin is reported to exhibit both p53-dependent and -independent anti-tumorigenic activities, while NAG-1 and p21 serve as the target genes for this flavone. Apigenin may induce pro-apoptotic proteins (NAG1 and P53) and the cell cycle inhibitor (p21). Additionally, apigenin has been shown to lower the number of polyps in animal models, where an increase in p53 expression was observed [67]. Apigenin has also been proven useful as an adjuvant therapy with 5-FU, thereby reducing acquired 5-FU resistance through inhibiting Thymidylate synthase (TS) expression. Furthermore, the molecule can enhance P53 expression, which leads to ROS generation and dysregulation of Ca^2+^, thereby arresting the cell cycle and enabling depolarization of the mitochondrial membrane potential (MMP) in colon cancer cells [68]. The anticancer mechanism of Apigenin in HCT116 was also unveiled through transcriptome studies, which identified the crucial role of miRNA-215-5p in the apigenin-mediated anticancer mechanism. The observed upregulation of miRNA215-5p was linked to regulating transcription factors in the E2F family, leading to cell cycle arrest in the G0/G1 phase [66]. Apigenin also inhibited the translocation of β-catenin into the nucleus (Figure 3), thereby downregulating downstream gene expression in a dose-dependent manner [69]. Even though the mechanism remains unclear, apigenin may exhibit anti-tumor effects in a manner dependent on the gut microbiota. The investigation highlighted the ineffectiveness of apigenin on the general health of the mice after depleting the gut microbiota through antibiotic treatment [70].

### 3.6. Quercetin

Quercetin is a plant flavonoid that is proposed to have anticancer effects. Its supplementation has been found to induce apoptosis, cell proliferation, and histopathological changes in colon cancer [71,72]. In vitro, the inhibitory effect of Quercetin on NF-κB pathway (Figure 3) was investigated in human colon cancer cell lines Caco2 and SW-620, and the results indicated a reduction in NF-κB DNA binding activity as well as dephosphorylation and upregulation of IκB-α. Further, there was an upregulation of pro-apoptotic proteins (Bax) and a decline in B-cell lymphoma 2 (Bcl-2) family, pointing to the mechanism that could be the induction of mitochondrial dysfunction and cellular apoptosis [73]. Inhibition of cell growth through apoptosis induction was observed in the CT-26, PC-12, LNCaP, and PC-3 cancer cell lines, and the in vivo reduction of CT-26 and MCF-7 tumor volume in mouse models was also achieved [74]. The unabsorbed flavonoids undergo metabolism and catabolism by gut microbiota in a time-dependent manner, yielding various phenolic catabolites, including phenylpropanoic, phenylacetic, and benzoic acid derivatives [75].

### 3.7. Curcumin

Curcumin is a well-documented polyphenol with reported antioxidant, anti-inflammatory, and anti-cancer activities. The compound exhibited in vivo inhibition of CRC progression via CD8+ T cell infiltration and ferroptosis. Curcumin could enrich the SCFA-producing Lactobacillus and Kineothrix, and surprisingly, FMT (Fecal microbiota transplant), followed by depletion of microbiota through antibiotic treatment, further emphasized that curcumin could alleviate CRC symptoms in a gut microbiota-dependent manner [76]. Curcumin is also proposed to be capable of regulating epigenetic machinery and is found beneficial in reducing the polyp number and size [77].

### 3.8. Other Anticancer Polyphenols

A pectin polyphenol complex derived from alperujo, a by-product of olive oil production, has an antiproliferative effect on Caco2 carcinoma cells. The extract could downregulate the production of pro-inflammatory cytokines like TNF-α, IL-1β, and IL-6 in LPS-stimulated murine peritoneal macrophages and also reduce ROS and nitrite in these cells [78]. Numerous reports emphasize the anticancer and antitumorigenic potential of various polyphenols; however, a detailed discussion of each compound is beyond the scope of this article. Some of the reported compounds are summarized in Table 1.

## 4. Dietary Polyphenols in Modulating Other Gut Microbial Metabolites in CRC

Bile acids are produced during cholesterol metabolism, which is believed to promote CRC, and the increased fecal levels of secondary bile acids are concerning. Research evidence suggests that the synthesis of bile acids and the regulation of oncogenic signals by bile acids and their metabolites are worth exploring in the context of CRC prevention and treatment [96]. Bile acids possess antimicrobial properties that can influence the composition of the gut microbiota, potentially contributing to dysbiosis. In healthy individuals, these effects are counteracted by commensal microbial populations capable of bile acid biotransformation, thereby preserving microbial and metabolic equilibrium [97]. A prominent example is *Clostridium scindens*, a fecal-derived bacterium capable of converting primary bile acids into deoxycholic acid (DCA), a secondary bile acid implicated in promoting CRC in murine models. DCA has been shown to impair CD8^+^ T cell-mediated antitumor immunity, thereby facilitating tumor progression. Targeted depletion of *C. scindens* through bacteriophage therapy has demonstrated potential in reestablishing immune surveillance and suppressing tumor growth, highlighting the critical immunomodulatory influence of bile acid metabolism in CRC pathogenesis [26]. Epidemiological data provide additional support for this mechanism; notably, the elevated incidence of CRC among Alaska Native populations has been partially linked to a low-fiber, high-fat dietary pattern, which diminishes colonic butyrate concentrations while enhancing exposure to deleterious bile acids such as DCA [43]. In this context, dietary polyphenols have emerged as promising modulators of gut microbiota composition and bile acid metabolism. Grape polyphenol (GP) supplementation in an animal model could alter their fecal microbiome profile by reducing bacteria associated with secondary bile acid accumulation, highlighting the importance of dietary polyphenol-based intervention strategies in human metabolic health. Moreover, polyphenols have been reported to interact with the bile acid–farnesoid X receptor (BA–FXR) signaling axis, modulating not only bile acid synthesis but also downstream metabolic and immunological pathways, including those involved in glucose regulation and inflammatory responses [98]. This highlights the therapeutic potential of dietary polyphenols in CRC prevention through modulation of microbiota and metabolic signaling pathways.

Research findings support the link between tryptophan (TRP) metabolism and CRC progression. Tumor cells utilize TRP to create a tumor microenvironment that promotes CRC, and gut microbial dysbiosis may disrupt TRP metabolism. Probiotics in the gut microbiome repair intestinal barriers and inhibit CRC progression through their metabolites and ability to regulate TRP metabolism [99]. This metabolic shift can result in either tumor-promoting or protective metabolites, contingent upon the composition and enzymatic activity of the gut microbiota. Dietary polyphenol luteolin acts as a complementary activator with gut microbial TRP metabolites in modulating the activation of aryl hydrocarbon receptor (Ahr), a critical role player in gut immunity and barrier host-microbe homeostasis [100]. These protective effects may be attributed to the Ahr-mediated release of IL-10 and IL-22, which contribute to epithelial barrier integrity and immunity [101]. Among TRP catabolites, indole derivatives synthesized by commensal bacteria are key activators of AhR signaling, promoting the expression of genes involved in maintaining epithelial barrier integrity, mediating anti-inflammatory responses, and exerting tumor-suppressive effects [102]. Accordingly, modulation of the gut microbiota to enhance the abundance of indole-producing bacterial strains, via probiotic supplementation or polyphenol-enriched dietary interventions, constitutes a promising approach for preserving intestinal homeostasis and mitigating the risk of CRC. Enriching probiotics through diet by utilizing TRP gut microbial metabolites shows promise for diet-based cancer management strategies.

## 5. Prebiotic Potential of Polyphenols in CRC

Prebiotics serve as supplements or food for beneficial bacterial inhabitants; adding these compounds enables the selective enrichment of beneficial bacteria. Probiotic bacteria can utilize these prebiotic supplements, and their biotransformation may produce value-added products that can either synergistically or antagonistically affect other gut microbes or host cells. Increasing reports on microbiome-host interactions underscore the importance of incorporating prebiotics into the gut ecosystem alongside gut microbiota [48,86,87]. Dietary polyphenols are a class of molecules that are gaining popularity in gut microbiome-based health management strategies. Although they are not classified as prebiotics, these molecules are attracting the attention of researchers [35,103]. Although various studies have highlighted the cellular, subcellular, and molecular mechanisms of different polyphenols in CRC, the potential for using these compounds as nutritional additives in therapy is limited. One of the major challenges associated with polyphenols is their limited bioavailability, which is influenced by various factors, including food processing, dietary composition, intake levels, interactions with other nutrients, distribution within the body, and environmental conditions. Recent studies emphasizing the biotransformation and bioavailability of these compounds have opened up a pathway for exploiting them in the nutraceutical industry as promising candidates, considering their impact on gut microbial metabolism. This biotransformation results in the generation of bioactive metabolites that can modulate host physiological processes and immune functions, thereby contributing to the prevention and therapeutic management of CRC. Despite proposals for using these compounds as prebiotics to enrich gut microbes and maintain good gut health based on various systematic reviews and meta-analyses, further clinical studies and exploration of the mechanisms are needed. The disease-alleviating and biocontrol effects of polyphenols in treating colonic diseases were tested in animal models, where experimental groups were fed polyphenol-rich grape powder. A supplementation of 10% GP-enriched *Lachnospiraceae* bacteria, known for butyrate production, resulted in a two-fold increase in butyrate levels in healthy animals. However, the elevated butyrate levels were not significant in the diseased group. Similarly, the diet could alleviate dextran sulfate sodium-induced colitis in the administered groups. In contrast, this protective effect was not observed in antibiotic-treated animal groups, suggesting an interplay between a polyphenol-rich diet and the colonic microbiota [104]. A polyphenol-rich cinnamon bark extract, upon in vitro digestion, produced cinnamic acid, which enabled the enrichment of probiotic strains. Additionally, the extract exhibited antitumor activity on the SW480 cell line, which is maintained upon probiotics fermentation [105]. Similarly, polyphenolic compounds, particularly those belonging to anthocyanins, were isolated from the fruits of *Vaccinium vitis-idaea* L. (lingon berries) in Bulgaria. The prebiotic potential of the lyophilized lingonberry extract was evaluated by exploring the metabolic profile of the probiotic strain *Lactobacillus plantarum* L10 at varying extract concentrations. The study reinforces that polyphenol extracts enhance the fermentation rate, leading to increased levels of SCFAs, such as acetate and butyrate, in the metabolite pool [106]. Olive oil leaf extracts underwent in vitro fermentation, mimicking colon fermentation, and produced enhanced levels of acetate, propionate, and butyrate acids compared to the positive control (fructose oligosaccharides), promoting the enrichment of beneficial bacteria such as *Bifidobacterium* spp. and *Clostridium leptum*. The major phenolic compounds identified after fermentation were hydroxytyrosol and tyrosol, which may contribute to the enrichment of beneficial bacterial growth [107]. These findings highlight the potential of olive leaf-derived polyphenols to modulate the gut microbiota beneficially, promote the production of health-associated SCFAs, and contribute to the maintenance of intestinal health [108]. A native berry from Brazil, Jabuticaba [*Myrciaria jaboticaba* (Vell.) O.Berg], which is a rich source of polyphenols such as ellagic acid and anthocyanins, was analyzed for its ability to modulate gut microbiota in carcinogenesis induced in Wistar rats. The rats were fed yogurt mixed with Jabuticaba extract (LJE), which was found to diversify the rat colon microbes in fecal microbiome analysis, thus supporting the in vivo prebiotic effects of polyphenolic compounds [109]. Recent studies and evidence suggest that polyphenols can elevate the beneficial bacterial population in the microbiome and reverse disease-induced effects of dysbiosis [27]. To establish this fact, more studies incorporating novel omic techniques with microbiome analysis, assisted by gnotobiotic mice, are needed [27,110]. Such integrative approaches will provide a more comprehensive understanding of the complex interactions among polyphenols, the gut microbiota, and host physiology, thereby informing the development of targeted dietary strategies for the prevention and management of CRC. Human-colonized microbiomes fed matcha green tea and were subjected to metabolomic analysis of green tea and plasma. The study revealed strong associations between green tea compounds in plasma and specific gut bacteria, underscoring the importance of dietary intake and gut microbiota [110]. Similarly, the dietary intake of quercitrin, a glycosylated dietary plant flavonoid, has been found to alter the colon metabolite pool and microbiome in mice. At the same time, an increase in the content of SCFA was also observed [111]. A fecal microbiome supernatant from apigenin-fed mice, when transplanted into antibiotic-treated mice, could regain its antitumor effect, highlighting that tumor carcinogenesis can be modulated through dietary supplements that modify gut flora [70]. Similarly, the intraperitoneal injection of tyrosol, combined with the intragastric administration of *Faecalibacterium prausnitzii*, resulted in an elevated level of CD8+ T cells in the blood, indicating an enhanced immune response within the tumor microenvironment. This suggests the potential prebiotic properties of tyrosol in modulating CRC tumor immunity [63]. Collectively, these studies highlight the multifaceted roles of dietary polyphenols in shaping gut microbiota composition and function, stimulating SCFA production, and modulating immune responses, thereby underscoring their potential in colorectal cancer prevention and treatment. Literature research and the published data emphasize the health benefits of dietary phenols and their potential role in improving gut barrier functions, modulating bile acids, and influencing gut immunoglobulins, thereby reducing inflammation. However, challenges remain in establishing this heterogeneous group of compounds in real-time applications.

## 6. Challenges in Administering Dietary Polyphenols as Prebiotics for CRC Management

The current surge in cancer incidence, increasing reports on drug resistance, as well as the high cost and side effects of therapeutic drugs, highly promote the research on nutraceutical-based cancer management therapies [112]. Dietary polyphenols are considered a beneficial choice due to their widespread presence in plant-based foods, their established potential to stimulate beneficial bacteria, and their inhibitory effect on pathogenic microbes [113]. Investigations and emerging evidence suggest using various natural polyphenol-rich compounds as dietary prebiotic supplements, as they can reverse dysbiosis, protect gut barrier integrity, and be effective in modulating CRC progression [3,104,114]. Nonetheless, translating these findings into clinical applications remains challenging, largely due to the intricate and dynamic interplay among polyphenols, the gut microbiota, and host-specific factors. The use of these dietary compounds as prebiotics, in combination with probiotics, has been proposed as an emerging adjunct therapy in CRC management; however, real application is still needed to overcome many hurdles. The clinical application of bioactive compounds and metabolites in CRC treatment is limited. Certain phytochemicals, including curcumin and resveratrol, have been investigated in clinical trials due to their ability to manage cancer. Still, the results have been inconsistent, attributed to fluctuations in personalized outcomes and poor bioavailability [115]. The therapeutic efficacy of polyphenols is often limited by their poor bioavailability, which is attributed to factors such as low solubility, instability within the gastrointestinal environment, and rapid metabolic degradation. Bioavailability may be enhanced through enzyme-assisted extraction, encapsulation, chemical modification, and co-administration with probiotics to ensure stability of bioactive compounds and facilitate their biotransformation by gut microbes [30]. Encapsulation techniques and nano delivery systems further enable prolonged therapeutic effects, thereby facilitating long-term disease management [116]. Recent advancements in nanotechnology have facilitated the development of innovative delivery systems, including mesoporous silica nanoparticles and lipid-based carriers, designed to protect polyphenols from gastrointestinal degradation and improve their targeted delivery to the colon. Further research has focused on enhancing bioavailability, including chemical modification, the application of novel delivery systems, and leveraging AI (artificial intelligence)-based delivery systems to improve human health [30,116]. Gut microbial metabolism is complex, and gut microbes are unique and diverse among individuals, varying by anatomical location, lifestyle, and genetics [117,118]. Further, polyphenols vary in their distribution of bioactive principles, as well as in their absorption and metabolism among different individuals or ethnic groups [117]. The concentration of these molecules and their metabolites in cells is low; therefore, a high dosage of these compounds may be detrimental to the cells. Additionally, co-administering these compounds with conventional drugs may interfere with their absorption and effectiveness [35]. Personalized nutritional strategies that account for individual variations in microbiome composition and genetic background are critical for maximizing the efficacy of polyphenol-based interventions. Adequate research on toxicity, dosage, and interactions with conventional drug candidates is necessary. Therefore, more precise research on the individual’s microbiome profile and physiological functions is warranted. Research exploring computational prediction models that incorporate AI and machine learning (ML) may be employed to obtain gut microbiome readouts of various host variables, such as genetics, exercise, age, sex, diet, inflammation, and test results [22]. However, this is a time-consuming and costly effort. Yet, it is a research area worth exploring. The selection of specific probiotic microorganisms that align with the host’s health status, combined with prebiotics, produces metabolites of interest for regulating the immune response, showing promise for a gut microbial intervention strategy [119]. The use of microbiota-derived metabolites as biomarkers for CRC risk and as therapeutic agents is a promising area of research. Such targeted studies on prebiotic-probiotic combinations and their impact on the tumor microenvironment further underscore the need for in-depth investigations into their molecular mechanisms. The integration of multi-omics approaches—encompassing genomics, transcriptomics, proteomics, and metabolomics—offers a holistic framework for elucidating the complex interactions among polyphenols, gut microbiota, and host physiology, thereby advancing the development of effective strategies for CRC management. Although the influence of polyphenols on CRC development and progression is well-documented, the specific mechanisms of their action remain poorly understood, particularly regarding the preservation of beneficial gut microbial flora [120]. Further studies should uncover how these dietary molecules interact with the host microbiome and tumor microenvironment. This should be achieved with the support of proteomic, transcriptomic, and metabolomic studies, as well as FMT approaches [27,70,121]. While polyphenols show promise in CRC management, their clinical application is further limited by regulatory constraints. This extensive research explores the prebiotic potential of various polyphenolic molecules and their gut microbial metabolites. It incorporates intestinal microbes and CRC markers in vitro and in vivo investigations. It involves high-throughput molecular techniques and focuses on different probiotic-prebiotic formulations and personalized prebiotic/symbiotic interventions, which may provide new insights into microbiota-based cancer therapeutics, helping to navigate the regulatory environment to support their clinical applications.

## 7. Conclusions

Extensive research on dietary polyphenols and their bioactive potential has highlighted their vast bioactive potential, particularly in the context of CRC. Emerging research reports suggest that the gut microbiome plays a crucial role in converting polyphenols into a more biologically active form with enhanced bioavailability. These microbial biotransformations further improve their therapeutic potential and open avenues for employing these compounds as natural alternatives or synergistic additives to conventional drugs. However, despite these promising findings, critical mechanistic gaps persist; bridging these gaps is essential to translate the polyphenol-microbiome axis into effective personalized clinical care within the next decade. The complex interactions between gut microbiota and colon epithelium in CRC are poorly understood, and the precise molecular mechanisms involved in the interactions between gut microbial metabolites and epithelial cells in CRC must be unveiled. Moreover, the unique microbiome profiles of individuals, along with limited understanding of how specific polyphenols function as prebiotic modulators to shape a beneficial microbiome profile, pose significant challenges to generalizing polyphenol-based interventions. To fully harness the clinical potential of dietary polyphenols and the gut microbiome, Microbiome Avenue requires integrative approaches that combine metabolomics, metagenomics, and computational modeling. The integration of Al and ML will accelerate the progress by identifying predictive biomarkers and allowing precision-based personalized nutraceutical intervention in CRC management.

## Figures and Tables

**Table 1:** Dietary polyphenols and their gut microbial metabolites in CRC.

Dietary Polyphenol	Natural Source	Gut microbial Metabolites	Mechanism of Action
Genestein	Soy foods and legumes [79]	Dihydrogenistein, 6′-hydroxy-O-desmethylangolensin (6′-OH-O-DMA), 5-hydroxyl-equol (5-OH-equol) [80]	Modulation of gut microbs and their metabolites [81,82,83]
Luteolin	Celery, parsley, broccoli, onion leaves, carrots, peppers, cabbages, apple skins, and chrysanthemum flowers [84]	Eriodictyol, chalcone and dihydrochalcone [85]	Down regulation of matrix metalloproteinase (MMP)-2 and MMP-9, Modulation of intestinalmicrobiota [86], Modulating pleiotrophin (PTN) via miR-384 expression [87].
Rutin	Passion flower, buckwheat, tea, and apple [88]	Quercetin-3-glucoside and quercetin [89]	Upregulation of Akt, JNK1/2, and FOXO3a; Downregulation of AMPK [90,91]
Anthocyanins	Fruits, vegetables and cereals [92]	Protocatechuic acid, ferulic acid, gallic acid, vanillic acid and syringic acid and phloroglucinol aldehyde [92]	Gut microbial balance and maintains epithelial barrier integrity [92,93]
Silymarin	Milk thistle [94]	Multiple flavonolignan and flavonoid metabolites [95]	Inhibiting cell proliferation and upregulating apoptotic index [94]

## Data Availability

No new data were created or analyzed in this study. Data sharing is not applicable to this article.

## Abbreviations

AI	artificial intelligence
CRC	colorectal cancer
DCA	deoxycholic acid
EGFR	Epidermal growth factor
FMT	fecal microbiota transplant
GI	gastrointestinal
GP	grape polyphenol
GPR	Gprotein coupled receptor
HDAC	histone deacetylases
HT	hydroxytyrosol
IBD	inflammatory bowel disease
IBS	irritable bowel syndrome (IBS)
LPS	lipopolysaccharides
MD	mediterranean diet
ML	machine learning
MMP	metalloproteinase
ROS	reactive oxygen species
SCFAs	short-chain fatty acids
TME	tumor microenvironment
TRP	tryptophan
Tyr	tyrosol
UA	urolithin A
